# A Founder Mutation in *VPS11* Causes an Autosomal Recessive Leukoencephalopathy Linked to Autophagic Defects

**DOI:** 10.1371/journal.pgen.1005848

**Published:** 2016-04-27

**Authors:** Jinglan Zhang, Véronik Lachance, Adam Schaffner, Xianting Li, Anastasia Fedick, Lauren E. Kaye, Jun Liao, Jill Rosenfeld, Naomi Yachelevich, Mary-Lynn Chu, Wendy G. Mitchell, Richard G. Boles, Ellen Moran, Mari Tokita, Elizabeth Gorman, Kaytee Bagley, Wei Zhang, Fan Xia, Magalie Leduc, Yaping Yang, Christine Eng, Lee-Jun Wong, Raphael Schiffmann, George A. Diaz, Ruth Kornreich, Ryan Thummel, Melissa Wasserstein, Zhenyu Yue, Lisa Edelmann

**Affiliations:** 1 Department of Genetics and Genomic Sciences, Icahn School of Medicine at Mount Sinai, New York, New York, United States of America; 2 Department of Molecular and Human Genetics, Baylor College of Medicine, Houston, Texas, United States of America; 3 Department of Neurology, The Friedman Brain Institute, Icahn School of Medicine at Mount Sinai, New York, New York, United States of America; 4 Departments of Anatomy/Cell Biology and Ophthalmology, Wayne State University School of Medicine, Detroit, Michigan, United States of America; 5 Clinical Genetics Services, New York University Hospitals Center, New York, New York, United States of America; 6 Department of Neurology, New York University School of Medicine, New York, New York, United States of America; 7 Neurology Division, Children's Hospital Los Angeles, Los Angeles, California, United States of America; 8 Division of Medical Genetics, Children's Hospital Los Angeles, Los Angeles, California, United States of America; 9 Courtagen Life Sciences, Woburn, Massachusetts, United States of America; 10 Clinical Genetics Services, NYU Langone Hospital for Joint Diseases, New York, New York, United States of America; 11 Baylor Miraca Genetics Laboratories, Houston, Texas, United States of America; 12 Institute of Metabolic Disease, Baylor Research Institute, Dallas, Texas, United States of America; University of Washington, UNITED STATES

## Abstract

Genetic leukoencephalopathies (gLEs) are a group of heterogeneous disorders with white matter abnormalities affecting the central nervous system (CNS). The causative mutation in ~50% of gLEs is unknown. Using whole exome sequencing (WES), we identified homozygosity for a missense variant, *VPS11*: c.2536T>G (p.C846G), as the genetic cause of a leukoencephalopathy syndrome in five individuals from three unrelated Ashkenazi Jewish (AJ) families. All five patients exhibited highly concordant disease progression characterized by infantile onset leukoencephalopathy with brain white matter abnormalities, severe motor impairment, cortical blindness, intellectual disability, and seizures. The carrier frequency of the *VPS11*: c.2536T>G variant is 1:250 in the AJ population (n = 2,026). VPS11 protein is a core component of HOPS (homotypic fusion and protein sorting) and CORVET (class C core vacuole/endosome tethering) protein complexes involved in membrane trafficking and fusion of the lysosomes and endosomes. The cysteine 846 resides in an evolutionarily conserved cysteine-rich RING-H2 domain in carboxyl terminal regions of VPS11 proteins. Our data shows that the C846G mutation causes aberrant ubiquitination and accelerated turnover of VPS11 protein as well as compromised VPS11-VPS18 complex assembly, suggesting a loss of function in the mutant protein. Reduced *VPS11* expression leads to an impaired autophagic activity in human cells. Importantly, zebrafish harboring a *vps11* mutation with truncated RING-H2 domain demonstrated a significant reduction in CNS myelination following extensive neuronal death in the hindbrain and midbrain. Thus, our study reveals a defect in *VPS11* as the underlying etiology for an autosomal recessive leukoencephalopathy disorder associated with a dysfunctional autophagy-lysosome trafficking pathway.

## Author Summary

Genetic leukoencephalopathies (gLEs) are a group of heterogeneous disorders with white matter abnormalities in the central nervous system (CNS). Patients affected with gLEs have brain white matter defects that can be seen on MRI and exhibit variable neurologic phenotypes including motor impairment, hypotonia, pyramidal dysfunction, dystonia and/or dyskinesias, ataxia, seizures, cortical blindness, optic atrophy, and impaired cognitive development. The exact etiology of half of gLEs is unknown. We studied three unrelated families affected with an undiagnosed gLE and discovered a homozygous germline mutation c.2536T>G in *VPS11*, a gene involved in membrane trafficking and fusion of lysosomes and endosomes, as a novel cause of a new gLE syndrome. The mutation in *VPS11* results in protein instability and impaired protein complex assembly. In addition, we show that *VPS11* is required for proper autophagic activities in human cells. Importantly, we characterized a zebrafish line carrying a *vps11* mutation and confirmed its essential role in brain white matter development and neuron survival.

## Introduction

Genetic leukoencephalopathies (gLEs) are a group of heterogeneous disorders with white matter abnormality in the central nervous system (CNS) [1, 2]. Patients affected with gLEs manifest variable neurologic phenotypes including motor impairment, hypotonia, pyramidal dysfunction, dystonia and/or dyskinesias, ataxia, seizures, cortical blindness, optic atrophy, and impaired cognitive development [1, 3]. Currently, there are over 90 gLEs with primary or secondary white matter abnormalities which are inherited in dominant, recessive or X-linked forms [1, 2]. The genetic factors implicated in gLEs thus far suggest impaired activity in lysosomes, peroxisomes, mitochondria and intermediary metabolism [1]. However, much of the disease mechanism remains elusive and in at least half of individuals with a white matter disorder, the genetic etiology is unknown [4].

In this work, we sought to identify the genetic defects in five patients from three unrelated families affected with a previously unrecognized leukoencephalopathy disorder. Using whole exome sequencing, we identified a homozygous missense variant *VPS11*: c.2536T>G (p.C846G) in all five patients. Previous studies in yeast identified and characterized Vps (vacuolar protein sorting) proteins as key regulators of cargo delivery to vacuoles [5]. Severe growth, trafficking and organelle morphology defects were observed when Vps Class C proteins were mutated [6, 7]. Evolutionarily conserved from yeast to mammals [8], Vps-C core components were found in CORVET and HOPS protein complexes involved in membrane trafficking and autophagy, respectively [9]. Autophagy is a major catabolic cellular process for the degradation of proteins and organelles upon nutrients starvation. Impaired autophagy contributes to pathological conditions in multiple organs or tissues including neurodegeneration [10–13]. A combination of clinical evaluation, genetic analysis, biochemical and cellular assays, and an animal model study in this report demonstrates that the amino acid substitution, C846G, results in a loss of VPS11 function, and that VPS11 deficiency contributes to autophagy impairment, myelination defects and neurodegeneration underlying the pathogenesis of a novel gLE syndrome.

## Results

### Clinical Characterization

Patient A from Family I was born at full-term to non-consanguineous parents of Ashkenazi Jewish descent following an uncomplicated pregnancy and delivery. She came to medical attention at seven months of age for evaluation of delayed milestones. A clinical exam at that time revealed developmental delay and hypotonia, prompting a brain MRI which showed prominent lateral ventricles with scalloped borders secondary to decreased periventricular white matter volume and a diminutive corpus callosum. By two years of age, she had developed tonic-clonic seizures and was cortically blind and hearing impaired. At her most recent follow-up at eleven years of age, she was severely developmentally delayed, non-verbal, and non-ambulatory. She was primarily G-tube fed due to risk of aspiration and had a neurogenic bladder. Physical examination was remarkable for microcephaly (<2^nd^ percentile), non-dysmorphic facial features, diffuse hypotonia, joint contractures, and no hepatosplenomegaly. Comprehensive metabolic screening and genetic testing for chromosomal aneuploidy, Rett syndrome, and Fragile X were all normal. A younger sister (patient A-S1) was noted to have developmental delay at four months of age. An MRI at five months showed minimal myelination in the deep cerebellar white matter and a greatly diminished corpus callosum. A brother (patient A-S2) began displaying similar symptoms at two to three months old and had an MRI that was significant for white matter abnormalities. Both of siblings presented essentially the same phenotype at older ages as the proband.

**Fig 1:**
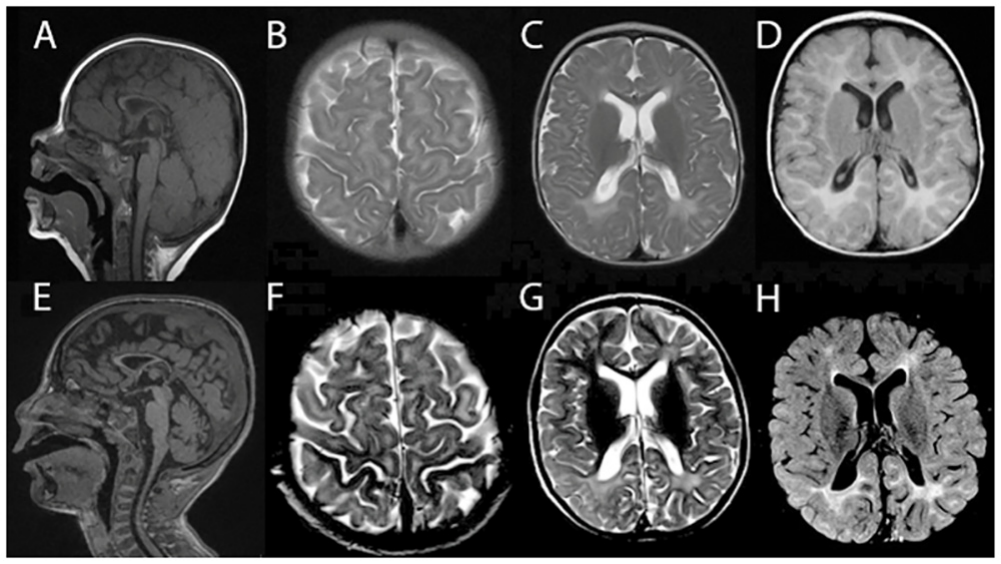
Brain MRI demonstrated white matter abnormality suggesting leukoencephalopathy in a patient homozygous for *VPS11*: c.2536T>G (p.C846G). In patient B at nine months of age, thin corpus callosum (A), T2 diffuse hyperintensity in the peri-Rolandic white matter (B) and posterior occipital white matter region (C) were seen; FLAIR diffuse hyperintensity in the white matter region was also seen (D). At five years of age, diminutive corpus callosum (E), T2 diffuse hyperintensity signal in peri-Rolandic areas (F) and supratentorial white matter, most pronounced within the bilateral parieto-occipital regions (G) were seen; FLAIR diffuse hyperintensity in the white matter region with a mild increase of myelination was seen(H).

Patient B from Family II was born at full-term to a non-consanguineous couple of Ashkenazi Jewish ancestry after an uncomplicated pregnancy and delivery. He was noted by his parents to have delayed milestones at five months of age. At most recent follow-up at five years of age, he was globally delayed, non-ambulatory and non-verbal, with cortical visual impairment and a history of febrile seizures. A swallow evaluation had demonstrated aspiration. Physical exam was notable for microcephaly (<2^nd^ percentile) and low weight (<5^th^ percentile), profound central hypotonia, mild lower leg hypertonia, joint contractures, dystonic posturing of the hands, and no hepatosplenomegaly. Brain MRI performed at nine months and five years of age showed multifocal T2/FLAIR hyperintense signal abnormalities of the supratentorial white matter extending from the periventricular regions into the juxtacortical regions, involving all lobes but most pronounced within the bilateral parieto-occipital regions. Diminished white matter volume with a hypoplastic corpus callosum was also noted. Relative to the nine month scan, the five year scan demonstrated a mild increase in myelination, however, a fairly extensive region of abnormal white matter including a diminutive corpus callosum was still evident on the five year scan consistent with a delayed myelination syndrome (Fig 1 and Table 1). Comprehensive metabolic screening and genetic testing including an Ashkenazi Jewish disease panel, dedicated mitochondrial gene microarray, karyotype, and chromosomal microarray were normal.

**Table 1:** Major leukoencephalopathy manifestations in five individuals from three AJ families with homozygous *VPS11*: c.2536T>G (p.C846G).

Patient	Onset and Initial Signs	Brain MRI	Cognitive Impairment	Hypotonia	Autonomic Dysfunction Signs	Seizure	Cortical Blindness or Optic Atrophy	Hearing Loss
A (10y,F)	4m (low tone, motor skill delay)	prominent lateral ventricle with scalloped margins, decreased volume of periventricular white matter, a small corpus callosum	+	+	oromotor dysfunction, neurogenic bladder	+	+	mild
A-S1 (9y, F)	6m (low tone)	minimal myelination and greatly diminished corpus callosum	+	+	N.A.	+	+	N.A.
A-S2 (7m, M)	3m (low tone)	minimal myelination	N.A.	+	N.A.	+	+	N.A.
B (5y,M)	6m (low tone, motor skill delay)	reduced periventricular white matter volume, diminutive corpus callosum	+	+	oromotor dysfunction, constipation	+	+	-
C (19y, M)	9m (low tone)	delayed myelination	+	+	oromotor dysfunction, constipation, cold and mottled extremities	+	+	mild to moderate

N.A., not assessed

Patient C from Family III was born at full-term to non-consanguineous parents of Ashkenazi Jewish heritage. Pregnancy was notable for an elevated alpha-fetoprotein measurement on maternal serum screening; prenatal ultrasound and amniocentesis were reportedly normal. He was born by Cesarean section for failure of labor to progress. He was noted by his parents to have strabismus and developmental delay as an infant. MRI at age 11 months showed periventricular white matter abnormalities in the occipital and frontal regions suggesting a myelination defect. At last follow-up at 19-years of age, he had profound intellectual disability, a history of static optic atrophy, mild to moderate hearing loss, oropharyngeal and GI dysmotility, and central dysautonomia characterized by sensitivity to temperature and mottling of the extremities. He was non-dysmorphic on exam and had low body weight (3rd-10th percentile), central hypotonia with spastic quadriplegia, and no hepatosplenomegaly. Prior extensive work-up to evaluate for an underlying mitochondrial disorder, including mitochondrial DNA analysis, had been non-diagnostic.

In summary, these five patients from three unrelated families of Ashkenazi Jewish ancestry exhibit highly concordant neurologic symptoms including severe developmental delay, seizures, hypotonia, cortical visual impairment or optic atrophy, absence of hepatosplenomegaly, and white matter abnormalities suggesting leukoencephalopathy. In addition, signs of autonomic dysfunction including oromotor dysfunction, body temperature instability, neurogenic bladder, and constipation were present in several of these patients (Table 1).

### WES Analyses

**Fig 2:**
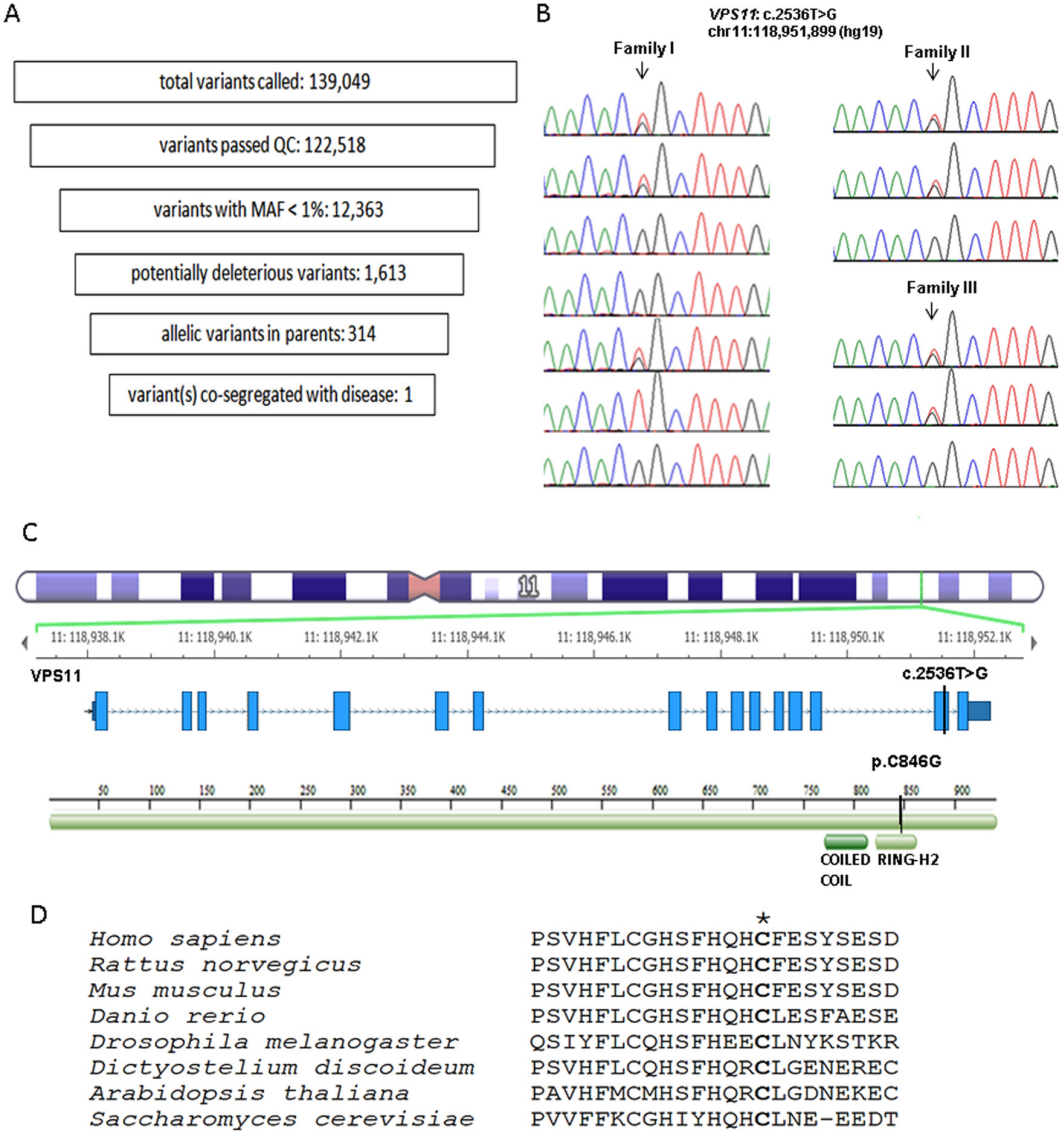
WES identified a homozygous mutation in the *VPS11* gene associated with gLE. (A) WES analysis filters applied to patient A’s familial sextet including parents and their two affected and two unaffected children. The number of variants passing each filter is indicated. (B) Sanger confirmation of the *VPS11*: c.2536T>G (p.C846G) variant (arrow) found in three families affected with gLE. In family I, from the top to the bottom are the genotypes at this locus in the parents, two affected children (patient A and A-S1), a carrier child, a non-carrier child and the 3rd affected child (patient A-S2). In family II and III, from the top to the bottom are the parents and their affected children (patient B and C). (C) The VPS11 C846 residue resides in a cysteine-rich RING-H2 domain in C-terminal region of VPS11. The genomic location of *VPS11*: c.2536T>G (p.C846G) and the amino acid change in the VPS11 protein are indicated by the black bars. The genomic structure of the *VPS11* gene illustration and the images are from Golden Helix GenomeBrowse visualization tool v2.0 by Golden Helix, Inc. (D) ClustalW 2.0 was used for multi-species alignment for VPS11 proteins. The mutation found in the patients results in an amino acid substitution in a cysteine residue (p.846C>G, denoted by *) conserved across species from yeast to human.

A total of 139,049 variants were identified by WES among the familial sextet in this index family (Family I) including the parents, two affected (patient A and A-S1) and two unaffected children (Fig 2A). After general filtering to exclude common (MAF>1%) and benign variants, a genetic filter assuming a recessive disease transmission mode in this family was applied. One homozygous variant *VPS11*:c.2536T>G (p.C846G) and two compound heterozygous variants *RGS22*:c.3007G>A (p.G1003R) and c.786C>A (p.N262K) co-segregated with the disease phenotype. Sanger sequencing revealed that the homozygous *VPS11*:c.2536T>G variant was also present in the third affected child (patient A-S2) who was not included in the WES analysis (Fig 2B). In addition, this affected child carried two copies of the *RGS22* WT allele; therefore, *RGS22* variants were ruled out as causative for the familial disorder. The *VPS11*:c.2536T>G variant is in exon 15 of the *VPS11* gene and results in a p.C846G missense change. The population frequency of this variant has not been reported in 1000 Genomes Database or the NHBLI Exome Sequencing Project (http://evs.gs.washington.edu/EVS/). In the ExAC database (http://exac.broadinstitute.org/), this variant has a very low minor allele frequency (0.00016 in non-Finnish Europeans, n = 67,740), and is not present in a homozygous state. The cysteine at position 846 of VPS11 is localized within a cysteine-rich RING-H2 domain (Fig 2C and 2D). The p.C846G change is predicted to be deleterious/damaging by SIFT, PolyPhen-2, GERP++, MutationTaster, and Mutation Assessor by in silico analyses. In an independent study, patient B and C were analyzed by WES using a different methodology [14] which did not yield any positive findings in known gLE genes. However, these two patients were found to carry the same homozygous variant *VPS11*:c.2536T>G identified in the index family.

### Carrier Frequency Analysis

Since all five patients have AJ ancestry, we were prompted to study the mutation frequency in this population. To this end, we carried out a Taqman assay using anonymized gDNAs from 2,026 healthy AJ individuals. Nine individuals were found to be heterozygous for this variant with no homozygotes identified, resulting in an allele frequency of 0.22% or 1:250 carrier frequency in this population. To examine whether the presence of the variant in this population is due to a founder effect, we searched runs of homozygosity containing the *VPS11*:c.2536T>G variant using the WES data sets from the index family. Next we genotyped five SNP markers (rs13929, rs470679, rs12795576, rs1135258 and rs10892350) in these three families and confirmed a minimum of 299 kb shared haplotype block (chr11:118915755–119215256) containing the *VPS11*:c.2536T>G variant in these families. All affected children were homozygous and the parent or sibling carriers were heterozygous for these SNPs. This finding supports that the *VPS11*:c.2536T>G variant represents a founder mutation in the AJ population.

### Functional Characterization of the *VPS11* Mutation

**Fig 3:**
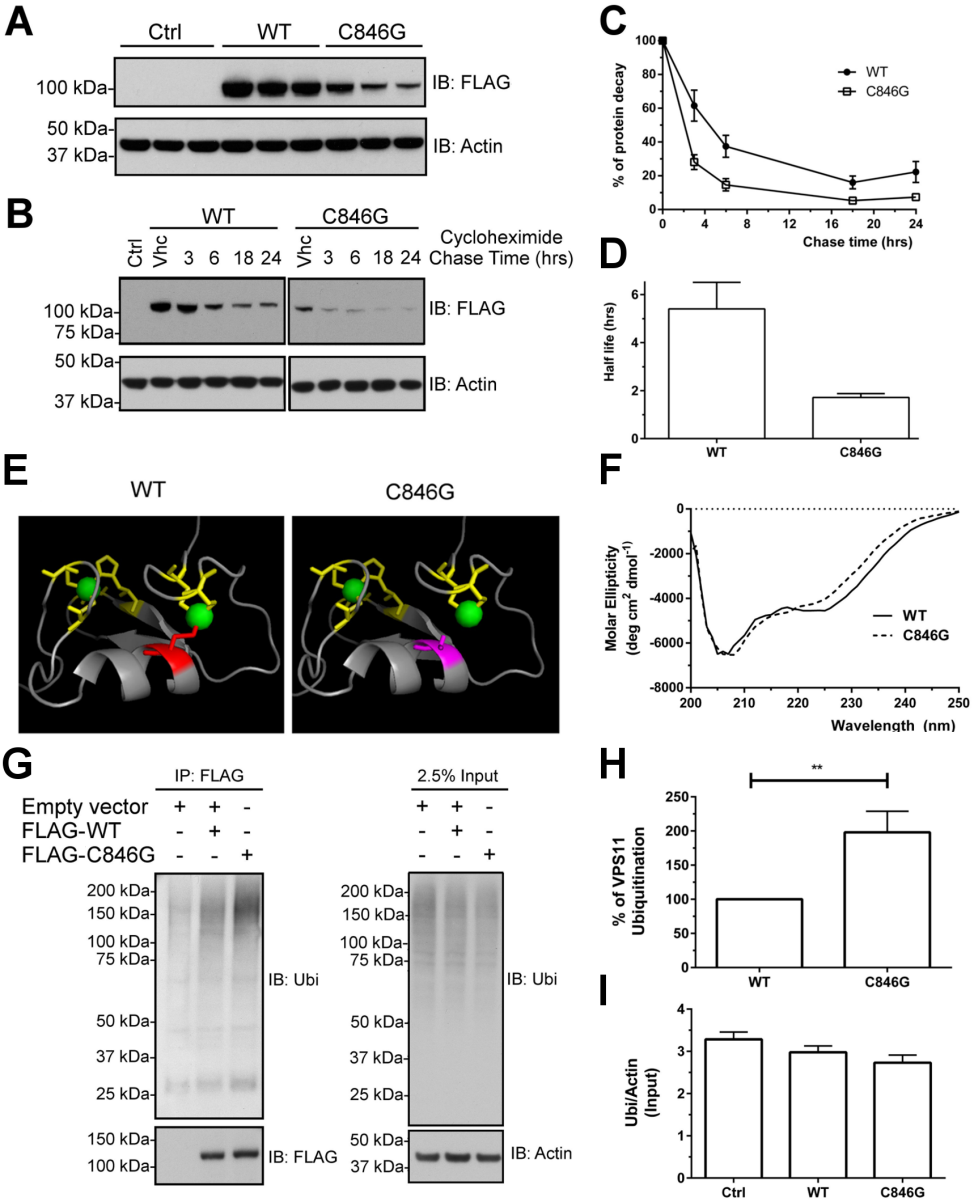
The VPS C846G mutation decreases the stability of VPS11 protein. HeLa cells were transfected with 0.5 ug DNA of empty vector (Ctrl), FLAG-tagged VPS11-WT or VPS11-C846G. (A) Expression of transfected plasmids was examined at 48 hrs post-transfection by Western blot analysis. (B) The chase started 24 hrs post-transfection and was allowed for the indicated time. Blots are representatives of four independent experiments. IB: Immunoblot. (C) The percentage of protein decay was graphically reported and the difference of the *in cellulo* half-life between the WT and C846 mutant of VPS11 was analyzed in (D). The statistical significance was determined by an unpaired student *t* test. Results are the mean±SEM of four independent experiments. (E) Images were prepared with PyMOL (www.pymol.org) using coordinates from the Protein Model Portal (VPS11_Q9H270, PDB 1iymA). (F) Far-UV CD spectra of the purified WT and C846G RING domain fragment proteins (amino acids 821–860). Results are the mean of three independent experiments. (G) HeLa cells were transfected with 2.0 ug DNA of empty vector, 1.0 ug of FLAG-tagged VPS11-WT with 1.0 ug of empty vector or 2.0 ug of FLAG-VPS11-C846G. Immunoprecipitations (IPs) were performed 48 hrs post-transfection and followed by Western blot analysis. Blots are representatives of four independent experiments. (H-I) The percentage of VPS11 ubiquitination was calculated and the difference of the *in cellulo* total ubiquitination profile was verified. The statistical significance was determined by an unpaired student *t* test. Results are the mean±SEM of four independent experiments. Data were considered significant when P values were <0.05 (*), <0.01(**) or <0.001 (***).

To determine the effect of the mutation on the VPS11 protein, we transiently expressed the FLAG-tagged wild-type (WT) VPS11 or C846G mutant in HeLa cells. Despite the same amount of transfected plasmid DNA, VPS11 protein harboring the C846G mutation had a remarkably reduced expression level compared to the WT protein (Fig 3A). To evaluate protein stability, we performed a cycloheximide chase assay in transfected cells. The half-life of the WT protein was five-fold higher than that of the C846G mutant (Fig 3B–3D). Homology modeling of VPS11-RING-H2 domain shows that the C846 residue is localized within the α-helix of this region that could be disturbed by the C846G mutation (Fig 3E). We purified Glutathione S-Transferase (GST) tagged fusion polypeptides corresponding to the RING-H2 domain of the WT and C846G variant (S1 Fig) and performed circular dichroism (CD) assay. Both WT and C846G RING-H2 domain fragments showed a typical curve for RING-H2 domain proteins with negative peaks at 206 nm and 225 nm (Fig 3F), suggesting that C846G does not disrupt the overall folding or secondary structure of the RING-H2 domain *in vitro*. Thus the instability of the C846G variant is unlikely due to improper folding of the RING-H2 domain. To determine the cause of the mutant protein’s instability *in cellulo*, we investigated the ubiquitination state of VPS11. We immunoprecipitated FLAG tagged VPS11 WT and C846G mutant and detected their ubiquitination levels using an anti-ubiquitin antibody. A significant increase in the levels of ubiquitination was observed in the mutant protein compared to the WT protein (Fig 3G and 3H). Given that RING-H2 domain containing proteins are known to possess E3-ubiquitin ligase activity [15], we examined the global ubiquitination level in above conditions and found no significant difference in the basal level of total ubiquitinated proteins (Fig 3G and 3I). Together, these results suggest that the increased ubiquitination of C846G mutant might result in accelerated degradation of VPS11.

**Fig 4:**
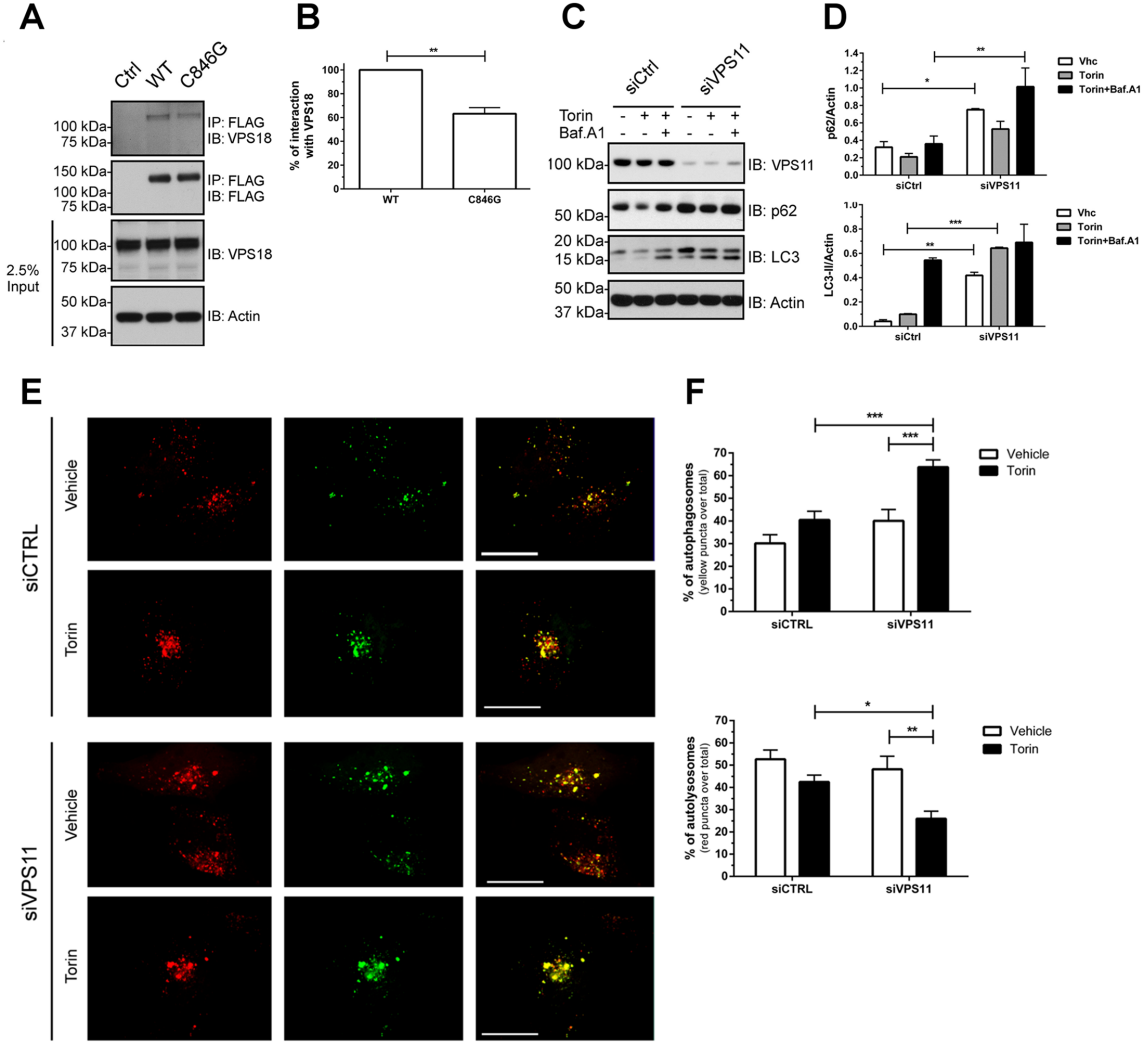
The p.C846G mutation impairs autophagy. (A) HeLa cells were transfected with empty vector (Ctrl), FLAG-tagged VPS11-WT or VPS11-C846G. Immunoprecipitations were performed 48 hrs post-transfection and followed by Western blot analysis. Blots are representatives of three independent experiments. (B) The densitometry values of co-immunoprecipitated endogenous VPS18 were normalized to the amount of immunoprecipitated FLAG-VPS11 WT or C846G and reported as the mean±SEM of three independent experiments. The statistical significance was determined using an unpaired student t test. (C) Cells were transfected with scrambled siRNA control (siCtrl) or siVPS11. Blot is representative of three independent experiments. (D) The densitometry values of endogenous p62 and LC3-II were normalized to the amount of actin and reported as the mean±SEM of three independent experiments. The statistical significance was determined using a Two-way ANOVA test followed by Sidak’s multiple comparisons test. (E-F) Cells were transfected with scrambled siRNA control (siCtrl) or siVPS11. Quantification of autophagosomes (yellow puncta) and autolysosomes (red puncta) was performed using Green and Red Puncta Colocalization Macro for Image J (D. J. Swiwarski modified by R.K. Dagda). Scale bars: 20 μm. Data are representatives of two independent experiments. Results are the mean±SEM of at least fifteen images. The statistical significance was determined using a Two-way ANOVA test followed by Tukey’s multiple comparisons test. Data were considered significant when P values were <0.05 (*), <0.01(**) or <0.001 (***).

Previous characterization of the architecture of the yeast CORVET and HOPS components suggested that VPS11 carboxyl-terminal region was crucial for the formation of both complexes [16–18]. Therefore, we investigated whether the C-terminus residing C846G variation affected VPS11-VPS18 interaction. Following the immunoprecipitation of the FLAG-tagged WT and C846G proteins, we found that the C846G mutation significantly decreased the interaction between VPS11 and the endogenous VPS18 (Fig 4A and 4B) suggesting that the C846G variant reduced the Vps-C core assembly.

As a component of HOPS complex important for endo-lysosomal trafficking, VPS11 has been implicated in the regulation of autophagy in the lower eukaryotic organisms [16, 19–22]. Given that C846G mutant displays protein instability and reduced complex formation with VPS18, we reason that C846G is a loss of function mutation. Thus we performed siRNA knockdown to investigate the consequence of reduced *VPS11* expression on autophagy in human cells. We found that knockdown of VPS11 caused accumulation of the autophagy markers, p62 and LC3 in basal condition (Fig 4C and 4D). Induction of autophagy through mTOR inhibitor Torin [23] promoted clearance of these markers in control cells (siCtrl) and had little effect in VPS11 depleted cells. No further increase of these markers was detected in si*VPS11* transfected cells treated with Bafalomycin A1 (autophagy flux inhibitor), suggesting that depletion of VPS11 does not affect a biosynthetic step, but rather diminishes the autophagy flux. Therefore, we performed autophagy flux assay using tandem fluorescent protein fusion RFP-GFP-LC3 reporter to evaluate the impact of VPS11 depletion on fusion between autophagosomes and lysosomes [24]. In the reporter plasmid transfected cells we observed significant accumulation of immature autophagosomes (red and green fluorescence merge, resulting in yellow) and reduced amount of autolysosomes (red only due to quench of green fluorescence in acidic vacuoles) in VPS11-depleted cells compared to control cells upon autophagy induction (Fig 4E and 4F). Altogether, the data demonstrate that reduced expression of VPS11 impaired autophagy flux in human cells and indicate that C846G is a loss-of-function mutation.

### Neuronal Cell Death and Myelination Defects in Zebrafish Mutant *vps11(plt)*

**Fig 5:**
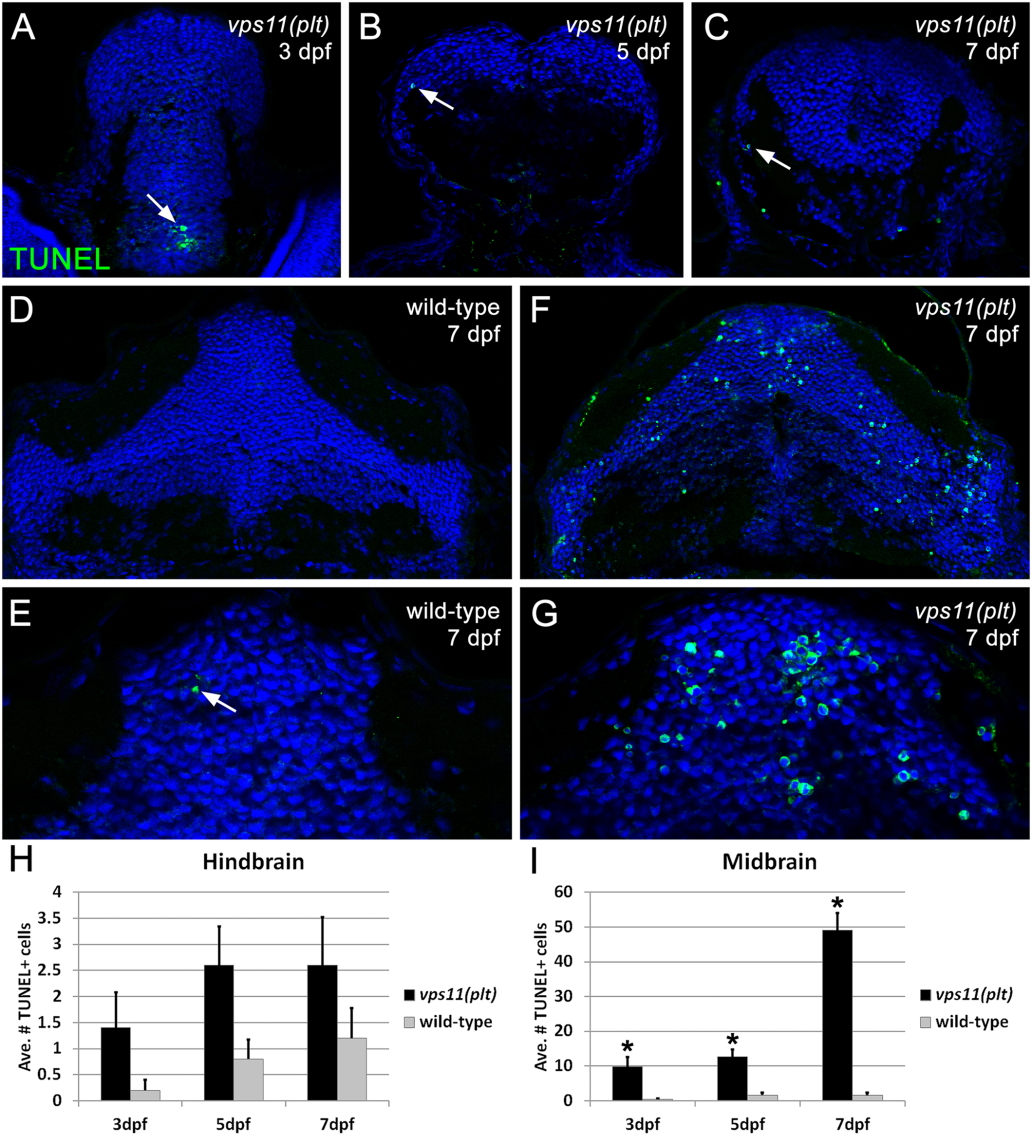
Significant cell death is observed in the CNS of zebrafish *vps11(plt)* mutants. TUNEL assay was performed on cryosectioned CNS tissue from *vps11(plt)* mutants and wild-type siblings at 3, 5, and 7 days post-fertilization (dpf). (A-C) Minimal apoptotic TUNEL+ cells were observed in the hindbrain of *vps11(plt)* mutants at 3, 5, and 7 dpf, respectively. (D-E) Tissue section of the midbrain of a wild-type control animal at 7 dpf showing minimal cell death (panel E, arrow). (F-G) Tissue section of the midbrain of a *vps11(plt)* mutant at 7 dpf showing extensive cell death. (H) Quantification of the average number of TUNEL+ cells observed in the hindbrain in *vps11(plt)* mutants and wild-type siblings at 3, 5, and 7 dpf. No significant differences were observed. (I) Quantification of the average number of TUNEL+ cells observed in the midbrain in *vps11(plt)* mutants and wild-type siblings at 3, 5, and 7 dpf. Asterisk indicates significantly different from control.

To further delineate the role of VPS11 in brain development and CNS myelination, we characterized a zebrafish mutant *vps11(plt)* in which the RING-H2 domain in the VPS11 protein was abolished [25]. We tested whether these animals showed defects in the CNS that were similar to the loss of human VPS11. First, we analyzed zebrafish *vps11(plt)* mutants for CNS neuronal cell death using TUNEL labeling at 3, 5, an 7 days post-fertilization (dpf). Compared with wild-type siblings, *vps11(plt)* mutants showed mild cell death in the hindbrain (Fig 5A–5C and 5H), but significant cell death in the midbrain (Fig 5F–5G and 5I). Immunolabeling of the active form of Caspase-3 [26, 27] confirmed that these cells were undergoing apoptosis (S3 Fig).

**Fig 6:**
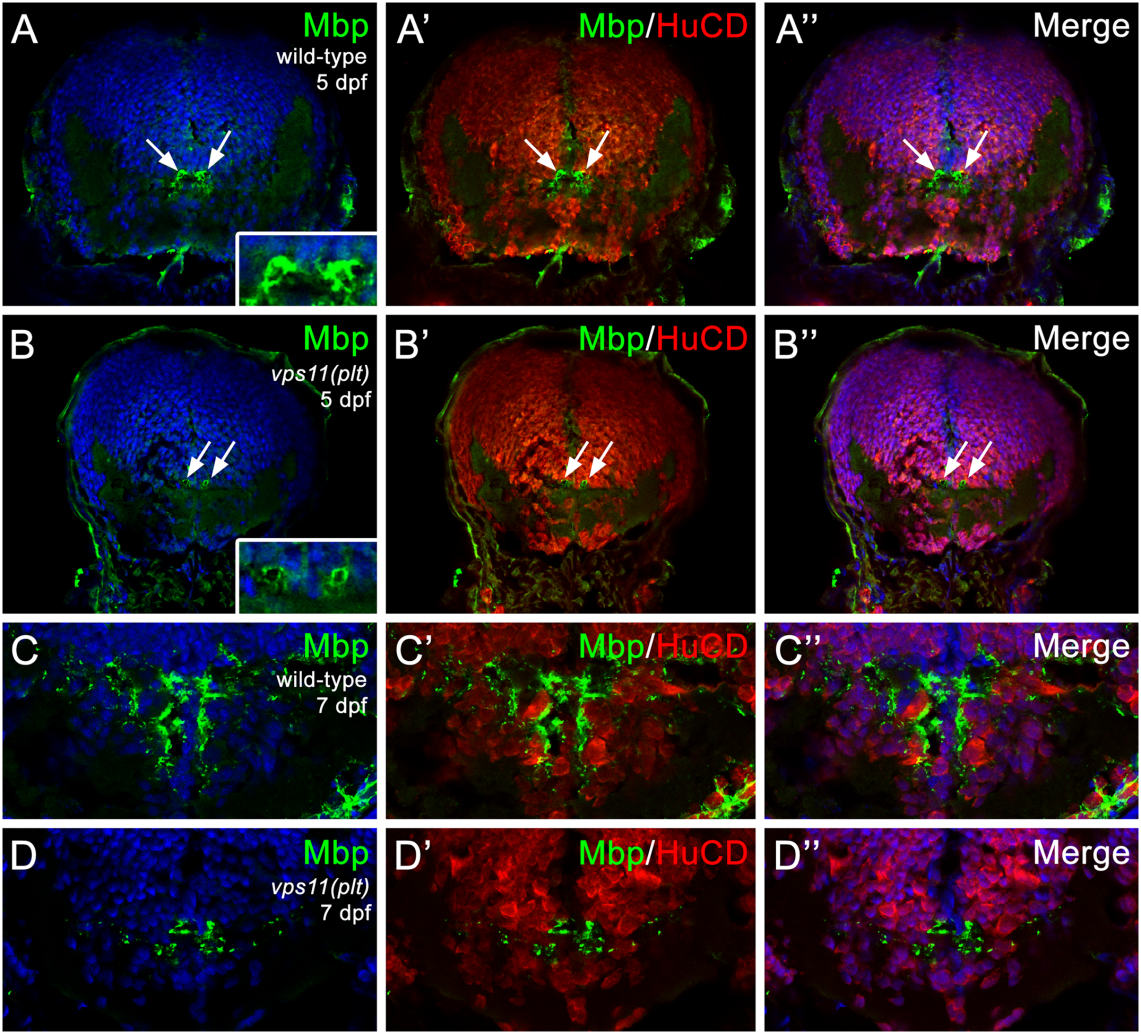
Progressive loss of myelination in zebrafish *vps11(plt)* mutants. Cryosectioned CNS tissue from *vps11(plt)* mutants and wild-type siblings were immunolabeled with Mbp (myelin; green) and HuCD (neurons; red) at 3 and 5 dpf. (A-A”) Mbp immunolocalization to Mauthner axons in the hindbrain of wild-type animals at 5 dpf (arrows). (B-B”) Mbp immunolocalization to Mauthner axons in the hindbrain of *vps11(plt)* mutants at 5 dpf (arrows). (C-C”) Mbp immunolocalization in the hindbrain of wild-type animals at 7 dpf. (D-D”) Significant reduction in Mbp immunolocalization in the hindbrain of *vps11(plt)* mutants at 7 dpf.

Next, we examined *vps11(plt)* mutants for defects in myelination, using immunolocalization of the major basic protein (Mbp) of myelin. Compared with wild-type siblings, *vps11(plt)* mutants showed a moderate reduction in Mbp immunolocalization in oligodendrocytes surrounding Mauthner axons in the hindbrain starting at 5 dpf (Fig 6A and 6B). However, the differences became much more pronounced at 7 dpf (Fig 6C and 6D). Quantification of the intensity of the Mbp staining showed a significant reduction in *vps11(plt)* mutants at 7 dpf when compared with wild-type siblings (38% of control level expression; p<0.05; N = 5, S2 Fig). Taken together, these data show that loss of VPS11 function in a zebrafish model results in CNS neuronal death and a significant reduction in myelination.

## Discussion

Our extended WES analysis on a familial sextet and two additional unrelated AJ families has linked a single variant *VPS11*: c.2536T>G (p.C846G) to a new autosomal recessive leukoencephalopathy syndrome with brain myelination defects, severe motor skill deficits, cortical blindness or optic atrophy, intellectual disability, and seizures. We show that the cysteine to glycine change, which occurs at an evolutionarily conserved cysteine rich RING-H2 domain in the C-terminus of VPS11, decreases its protein stability and enhances its ubiquitination levels, which are likely associated with accelerated protein turnover. Our study also demonstrates that VPS11 deficiency resulted in impairment of autophagy flux in human cells, which is in agreement with the results from the yeast and zebrafish studies [28, 29]. Furthermore, zebrafish harboring a *vps11* mutation with abolished RING-H2 domain exhibits reduced myelination and significant neuronal death, confirming an essential role for VPS11 in CNS development and substantiates the causality of the C846G mutation in leukoencephalopathy. Our study establishes that defects in *VPS11* are a novel cause of leukoencephalopathy, and identifies the C846G as an AJ founder mutation, thus providing valuable knowledge for genetic counseling in the affected families and in predicting disease risk. The diagnosis for VPS11 related leukoencephalopathy solely based on clinical exam or brain imaging can be challenging, but it should be suspected in a patient of Ashkenazi Jewish descent with infantile-onset leukoencephalopathy associated with severe developmental delay, visual impairment and seizures. The lack of a distinctive clinical phenotype is a common barrier to diagnosis in patients with leukoencephalopathy [1, 2], and underscores the need for comprehensive approaches to genetic testing (eg. panel-based testing, whole exome sequencing, or whole genome sequencing) in affected patients with nonspecific phenotypes [2]. These broad-based approaches also enable recognition of unanticipated phenotypic expansion and facilitate identification of novel disease genes.

VPS11 plays an important role in membrane trafficking and fusion of the lysosomes and endosomes by forming conserved protein complexes with a number of additional VPS proteins [16, 17]. Our study found that the VPS11-C846G is less stable than the WT protein. However, the altered stability does not appear to involve the misfolding of RING-H2 domain, but instead could be due to the increased ubiquitination of the VPS11 protein. Interestingly, RING domains usually exhibit an E3-ubiquitin ligase activity, which enables the protein to regulate its stability by inducing autoubiquitination [23, 30, 31]. Thus, future experiments should investigate the detailed mechanism by which C846G mutation enhances the ubiquitination of VPS11. Moreover, thorough investigation is required to determine whether proteasomal or lysosomal degradation is responsible for the turnover of VPS11 and how C846G variant affects the turnover in disease relevant cell types. Understanding the mechanism of increased ubiquitination is also crucial since E3-ligases represent potential therapeutic targets to modulate the ubiquitin-proteasome system activity, which may allow future treatment to regulate the proteome homeostasis. [18, 32, 33]. Finally, we found that the C846G mutation also significantly decreased the interaction between VPS11 and the endogenous VPS18 suggesting that the C846G mutation may affect the Vps-C core assembly.

Our study raises a question whether autophagic defects contribute to myelination defects as observed in the patients described in this study. Autophagy is known for the regulation of lipid metabolism and may have a role in myelination [34]. Knockdown of Vps16 (another component in Vps Class C) in *C*. *elegans* drastically reduced normal fat storage [35]. The observation highlights the importance of Vps-C complexes (HOPS and CORVET) in the regulation of lipid metabolism, an important process for myelination [36]. On the other hand, autophagy defects are associated with neurological disorders including early onset neurodegenerative disease [11–13]. Therefore, it is also possible that the myelination defects seen in our patients could be secondary to dysfunctional neurons as neuronal activity is essential for proper myelination *in vivo* [37]. A variety of leukoencephalopathies are neurodegenerative diseases with a prominent secondary white matter involvement such as GM1 gangliosidosis and neuronal ceroid lipofucinosis [1]. It is worth mentioning that in addition to motor symptoms commonly seen in primary leukodystrophies, microcephaly, severe cognitive impairments and seizures are present in our patients suggesting a primary neuronal/axonal defect in this disease. Interestingly, the zebrafish *vps11* mutant mode displays increased neuronal death in the midbrain and hindbrain. Notably, the neuronal death was observed at 3 and 5 dpf, which preceded the significant reduction in myelination observed at 7 dpf. Therefore, it is unlikely that the neuronal loss is due to myelination defects as seen in Pelizaeus–Merzbacher disease [38], but primary neuronal dysfunction and secondary deficit in myelination may explain the pathophysiology of this disease. Future experiments should also investigate neuronal or oligodendritic contribution to defective myelination or neuronal death using cell type specific deletion of the *VPS11* gene in mammals.

In summary, our study identifies a homozygous mutation in *VPS11* as causative in a novel leukoencephalopathy disorder associated with dysfunctional autophagy-lysosome trafficking pathway, and implicates VPS11 as a potential therapeutic drug target for this devastating infantile disease.

## Materials and Methods

### Ethics Statement

Informed consents of all patients enrolled in this study were obtained at Icahn School of Medicine at Mount Sinai or Baylor College of Medicine. The institutional review boards at Icahn School of Medicine at Mount Sinai or Baylor College of Medicine approved the study protocols. For the population screening of the *VPS11*: c.2536T>G (p.Cys846Gly) variant in the Ashkenazi Jewish population, peripheral blood samples were obtained with informed consent from individuals from the greater New York metropolitan area who requested carrier screen testing by the Mount Sinai Genetic Testing Laboratory. The screened population was composed of individuals who were reported to be 100% AJ.

### Statistics

Statistical analyses were performed using GraphPad Prism version 6.00 for Windows (GraphPad Software) using the unpaired Student’s t test and Regular Two-way ANOVA test followed by Sidak’s or Tukey’s multiple comparisons test. Data were considered significant when P values were <0.05 (*), <0.01(**) or <0.001(***).

### NGS Library Construction and Sequencing

DNA was extracted from peripheral blood using the PureGene Genomic DNA purification kit (Qiagen, Valencia, CA). Two micrograms of genomic DNA were fragmented by sonication to a peak sized at 250bp for each sample. NEBNext Ultra DNA Library Preparation Kit Illumina (Life Technology, Grand Island, NY) was used for DNA library preparation with custom ligation adapter according to the manufacturer protocol. AMPure XP beads (Danvers, MA, Beckman Coulter Genomics) were used for double size selection to achieve ~250 bp inserts. Each sample was then barcoded by eight cycles of PCR amplification after which three indexed samples were pooled into one capture reaction. Hybridization was performed using SureSelect Human All Exon V5 probe library (Agilent Technologies, Santa Clara, CA) and streptavidin-coated magnetic beads were added to the capture mixture for targeted isolation. The purified DNA was then amplified again by 11 PCR cycles. All captured libraries were quantified using the Agilent Bioanalyzer for normalization and underwent 2X100 bp paired-end sequencing in HiSeq 2500 (Illumina, San Diego, CA) using a high-throughput mode.

### Variant Calling and Analysis

Demultiplexed fastq data of each sample was processed using an in-house genome analysis pipeline (GAP) composed of bwa 0.7.5a, Picard 1.96, GATK 2.7, snpEff 3.0, BEDTools 2.16.2, and custom-developed software was used in parallel for variant calling confirmation. BAM files generated by this pipeline were used to visualize read pairs and variant calling in Integrative Genomics Viewer (Broad Institute, Cambridge, MA). Ingenuity variant analysis (Redwood City, CA) platform was used for select candidate variants based on variant frequency, pathogenicity, and inheritance filters.

### Sanger Sequencing for *VPS11*: c.2536T>G (p.C846G)

Purified DNAs were diluted to a concentration of 50 ng/μL. One microliter aliquot of the prepared DNAs (50 ng/μL) were then distributed to thin-walled PCR (0.2 mL) tubes with 1.2 μL of exon F/R primer mix (10 μM working solution), 15.4 μL distilled water, 2.5 μL 10X PCR buffer, 0.75 mM MgCl2, 4.0 μL 0.2 μM dNTP, and 0.2 μL Platinum Taq (5 U/μL).The following PCR profile was run: 95°C for 5’, (95°C for 30”, 60°C for 30”, 72°C for 30”) X 35, 72°C for 7’ and 4°C hold. Exo/SAP treatment was used to clean up the PCR products (2.5 μL Shrimp Alkaline Phosphatase and 1 μL Exonuclease). Reactions were processed in a thermal cycler programmed as follows: 37°C for 30’, 99°C for 15’, 4°C hold. Bi-directional DNA sequencing for *VPS11* exon 15 with 8–20 ng of the purified PCR product was conducted using procedures recommended by the manufacturer. PCR products were separated by electrophoresis in agarose gels to ensure proper amplification, which demonstrated a single strong band with the expected size for the exon to be analyzed.

### TaqMan Genotyping for *VPS11* Mutation

A TaqMan SNP Genotyping Assay (C__25755868_10) was ordered from Life Technologies (Carlsbad, CA) to detect the *VPS11*: c.2536T>G (p.C846G) variant. The DNA samples were not normalized prior to plating, but quantities fell within the suggested range of 1–20 ng. Samples were run on the GeneAmp PCR System 9700 (LTI) at the following setting: holds at 50°Cfor 2 min and 95°Cfor 10 min, and then 40 cycles at 95°Cfor 15 s and 60°Cfor 1 min. Allelic discrimination was performed on a LightCycler 480 (Roche Diagnostics Corporation, Indianapolis, IN).

### Reagents for Immunoprecipitation and Immunoblot

The monoclonal anti-FLAGM2 antibody (cat# F1804), the polyclonal anti-Vps18 (cat# SAB1105227) and Cycloheximide (cat# C-4859) was from Sigma. The polyclonal anti-Vps11 was from Bethyl Laboratories (cat# A303-528A). The monoclonal anti-β-Actin (clone 8H10D10) and anti-mouse IgG HRP-linked (cat# 7076S) antibodies were from Cell Signaling. The polyclonal anti-LC3 antibody was from MBL (cat# PM036). Polyclonal anti-p62 was from PROGEN (cat# GP62-C). The anti-guinea pig IgG-HRP antibody was from Santa Cruz Biotechnology (cat# sc-2903). The protease inhibitor tablets were from Roche Diagnostic (cat# 04693159001) and the phosphatase inhibitor tablets from Thermo Scientific (cat# 88667). The NuPAGE Novex 4–12% Bis-Tris protein gels were from Invitrogen (cat# NP0323BOX). Bafalomycin A1 was from CalBiochem (cat# 196000). Torin 1 was from Tocris Bioscience (cat# 4247). The polyclonal anti-ubiquitin was from Dako (cat# Z 0458). Dynabeads Protein G was from Novex (Life Technologies).

### Plasmid Constructs

The FLAG-Vps11-WT plasmid was prepared by PCR from the human cDNA clone template purchased from Dharmacon (cat# MHS6278-202759639). The PCR fragment was digested with NotI and XbaI and ligated into the p3XFLAG-CMV-7.1 expression vector from Sigma-Aldrich (cat# E7533). The FLAG-Vps11-C846G construct was generated using the QuikChange Lightning Site-Directed mutagenesis kit (Agilent Technologies) as recommended by the manufacturer. The GST-Vps11-RING-WT and GST-Vps11-RING-C846G were prepared by PCR from their respective FLAG-tagged templates. The PCR fragments were digested with EcoRI and XhoI and ligated into the pGEX-6P-1 from GE Healthcare (cat# 28-9546-48). The tandem RFP-GFP-LC3 plasmid was created and kindly provided by Tamotsu Yoshimori of Osaka University, Japan [24]. Integrity of the coding sequence of these constructs was confirmed by dideoxy sequencing.

### Cell Culture and Transfection

HeLa cells were maintained in DMEM (Dulbecco’s Modified Eagle’s Medium) (Gibco, Life Technologies) supplemented with 10% (v/v) FBS (Foetal Bovine Serum) at 37°C in a humidified atmosphere containing 5% CO2. Transient transfection of HeLa cells grown to 50–70% confluence were performed using the TransIT-LT1 Reagent (Mirus) according to the manufacturer’s instructions.

### Immunoblotting

HeLa cells were plated in 12-well plate at a density of 2× 10^5^ cells per well, transfected the same day with the indicated constructs and then maintained for an additional 48 h. The cells were then washed with ice-cold PBS and harvested in 50 μl of lysis buffer (150 mM NaCl, 50 mM Tris (pH 8.0), 0.5% Deoxycholate, 0.1% SDS 10 mM Na_4_P_2_O_7_, 1% IGEPAL, and 5 mM ethylenediaminetetraacetic acid (EDTA)) supplemented with protease and phosphatase inhibitor tablets. After 60 min of incubation in lysis buffer at 4°C, the lysates were then centrifuged for 15 minutes at 16 100 x g at 4°C. The protein concentration was determined using Pierce BCA Protein Assay Kit (Thermo Scientific). The cell lysates were denatured using 1X SDS sample buffer and analyzed by NuPAGE electrophoresis and immunoblotting with specific antibodies.

### Cycloheximide Chase Assay

HeLa cells were transfected with FLAG-tagged construct of WT and C846G protein. The chase has been performed as described in [39]. Briefly, 24, 42, 45 h post-transfection, culture medium was changed for the chase medium (DMEM supplemented with 20 mM HEPES). Cycloheximide was added to reach a final concentration of 100 μM. The 18 h chase conditions were start 24 h post-transfection and cells were harvested the following morning. Vehicle controls were 24 h treated with DMSO. At the required times, cells were washed with ice-cold PBS, collected, pelleted and flash-frozen in liquid nitrogen. Once all samples were frozen, cells were lysed as described previously and cell lysates were analyzed by NuPAGE and immunoblotting with the specified antibodies.

### Image Densitometry

Western blot quantification was meticulously performed using a procedure described [40] based on the recommendations of Gassmann et al.[41]. Specifically, all quantified immunoblots were revealed using the same type of films (HyBlot CL, Premium Autoradiography Film, cat# E3012, Denville Scientific, Inc.) and carefully exposed to avoid saturation. Films were scanned using a Epson Perfection v500 Photo scanner. Acquisition was performed at 600 dpi in 16- bits grayscale with auto-exposure and colour-correction options turned off. Images were analyzed using the ImageJ software. Lanes were selected and plotted using the ‘Gel analyzer’ functions. Peaks on the plots were individually closed to the background level of each lane using the Straight line’ tool and the enclosed area was measured using the ‘Wand’ tool. Results were compiled and reported as the mean±SEM of all quantified experiments.

### Recombinant Protein Production and Purification

The cDNA fragments coding for the Vps11-WT or C846G RING domain introduced in the pGEX- 6P-1 vector (Amersham Bioscience) were used to produce GST-fusion proteins in the OverExpress C41 (DE3) *E*.*coli* strain (Lucigen), which were purified using gluthation-Sepharose 4B (GE Healthcare) and the following buffer (10 mM Tris-HCl pH7.5, 150 mM NaCl, 1 mM EDTA, 0.02% reduced Triton-X-100 and 1 mM dithiothreitol). The GST tag was cleaved using Pierce HRV 3C Protease (Thermo Scientific, 88946) and the previous buffer. Purified recombinant proteins were quantified using Coomassie Plus Assay Kit (Thermo Scientific) and analyzed by NuPAGE electrophoresis followed by Colloidal Blue Staining Kit from Life Technologies.

### Circular Dichroism Spectroscopy

Far-UV (200–250 nm) CD experiments were performed at room temperature with a Jasco J-810 spectropolarimeter using a 1-mm path length quartz cuvette. Spectra of the WT and C846G RING domains were obtained for three different protein concentrations (95 uM, 75 uM, 60 uM) at a scan rate of 0.75 nm/s and a bandwidth of 1 nm. Each scan was performed two times for signal averaging. The CD signal was converted to mean residue molar ellipticity and the results from the three protein concentrations were averaged. Buffer for all CD experiments consisted of 10 mM Tris-HCl (pH7.5), 150 mM NaCl, 1 mM EDTA, 0.02% reduced Triton-X-100 and 1 mM dithiothreitol.

### siRNA Assays

The synthetic oligonucleotides ID J-007022-07 (siVPS11) targeting the human *VPS11* gene, and the negative control siRNA (siCtrl) (ON-TARGET plus Non-targeting Pool, catalogue Item D-001810-10-05) were purchased from Dharmacon. HeLa cells were transfected with 20 nm oligonucleotide using the Lipofectamine RNAiMax transfection reagent (Invitrogen) according to the manufacturer’s indications except for the following modifications: cells were seeded directly into the transfection mix at twice the cell density as indicated in the basic protocol. Protein expression analysis by Western blotting experiments were performed at 48 h post-transfection.

### Autophagy Assay

Autophagy has been induced using Torin1 (250 nM) and blocked with Bafalomycin A1 (100 nM) both diluted in fresh culture medium supplemented with 20 mM HEPES. DMSO was added in control condition (Ctrl). The cells were incubated at 37°C and the induction allowed for 2 h. The cell lysates were then analyzed by NuPAGE electrophoresis and immunoblotting with specific antibodies.

### Immunoprecipitation

Immunoprecipitations were performed as in [30]. Briefly, HeLa cells were transiently transfected with the indicated constructs and were maintained as described above for 48 h. The cells were then washed with ice-cold PBS and harvested in 300μl of lysis buffer (150 mm NaCl, 50 mm Tris (pH8.0), 0.5% deoxycholate, 0.1% SDS, 10 mm Na4P2O7, 1% IGEPAL, and 5 mm ethylenediaminetetraacetic acid (EDTA)) or ubiquitination lysis buffer for ubiquitination experiments (50 mm Hepes (pH7.5), 250 mm NaCl, 2 mm EDTA or 1 mm CaCl2, 0.5% IGEPAL, 1 mm PMSF, 1 mm NaF, 1 mm Na3VO4, 10% Glycerol and 10 mM N-ethylmaleimide). Both buffers were supplemented with protease inhibitor tablets. After 60 min of incubation in lysis buffer at 4°C, the lysates were then centrifuged for 15 min at 16100 x g at 4°C. The protein concentration was determined using Pierce BCA Protein Assay Kit (Thermo Scientific). 700–1000 μg of proteins was used for immunoprecipitations. One microgram of specific antibodies was added to the supernatant. After 3 hours of incubation at 4°C, 30 μL of Dynabeads Protein G was added, followed by an overnight incubation at 4°C. Samples were then centrifuge 1 min in a microcentrifuge and washed three times with 1 mL of lysis buffer, immunoprecipitated proteins were eluted by addition of 90 μL of 4X SDS sample buffer, followed by 5–10 min incubation at 95°C. Initial lysates and immunoprecipitated proteins were analyzed by SDS-PAGE and immunoblotting with specific antibodies.

### Immunofluorescence and Confocal Microscopy

HeLa cells were plated in 24-well plates containing coverslip coated with 0.1 mg/ml poly-L-lysine (Sigma), at a density of 3 × 10^4^ cells per well. The following day, the cells were transiently transfected with 20nM of siRNAs (siCTRL or siVPS11). 24 hrs after siRNA transfection, medium was changed and RFP-GFP-LC3 plasmid was transfected. The following day, cells were washed once with PBS and treat with DMSO or Torin1 (250nM). The cells were incubated at 37°C and autophagy induction was allowed for 2 h. Cells were then fixed immediately with 4% (v/v) PFA (paraformaldehyde) in PBS for 10 min. at room temperature (RT). Subsequently, the cells were washed three times with PBS for 5 min. at RT and the coverslips were mounted using ProLong Gold antifade reagent. Confocal microscopy was performed using a scanning confocal microscope (Zeiss LSM780) with a ×63 oil-immersion objective lens and images were processed using Image J software (NIH).

### Zebrafish Lines and Maintenance

Two zebrafish lines were used in this study: AB (wild-type) and *vps11(plt)* mutant embryos[25]. Fish were fed a combination of brine shrimp and dried flake food three times daily and maintained at 28.5°C on a 14 h light (250 lux): 10 h dark cycle[42]. All animal care and experimental protocols used in this study were approved by the Institutional Animal Care and Use Committee at Wayne State University School of Medicine.

### Immunohistochemistry and Confocal Microscopy of Zebrafish CNS Tissue

Wild-type and *vps11(plt)* mutants fish were euthanized at 3, 5, and 7 days post-fertilization (dpf) fixed in 9:1 ethanolic formaldehyde (100% ethanol: 37% formaldehyde) overnight at 4°C. The tissue was cryoprotected with washes in 5% sucrose/1XPBS at room temperature for 2 hours and 30% sucrose/1XPBS overnight at 4°C. Tissue was embedded in Tissue Freezing Medium (TFM, Triangle Biomedical Sciences, Durham, NC) and frozen at -80°C. Tissue was then cryosectioned in the transverse orientation at 16 mm, transferred onto glass slides, dried at 55°C for 2 hours, and stored at -80°C. Immunohistochemistry was performed as previously described[43]. Slides were incubated overnight at room temperature using the following primary antibodies diluted in blocking solution: rabbit polyclonal anti-myelin basic protein (Mbp) antisera (1:200, a kind gift from Bruce Appel), mouse monoclonal anti-HuCD antibody (1:50, Invitrogen, Grand Island, NY), rabbit anti-activated Caspase-3 (1:500, BD Biosciences, San Jose, CA). Secondary antibodies included AlexaFluor goat anti-primary 488 and 594 (1:500, Invitrogen) and nuclei were stained with DAPI. Cover slips were mounted using ProLong Gold (Molecular Probes, Eugene, OR).

Confocal microscopy was performed with a Leica TCS SP8 confocal microscope. Unless otherwise noted (eg. retina), images were obtained in the hindbrain region. All images were obtained using identical intensity settings for the Mbp immunodetection and identical parameters for obtaining a z-stack image (total thickness: 1.5 mm; slices: 4). Quantification of the difference between total Mbp immunofluorescence at 7 dpf was performed (n = 5 per group) using the Corrected Total Cell Fluorescence method [44].

### Cell Death Analysis

Terminal Transferase dUTP Nick End Labeling (TUNEL) assay was performed on frozen sections using the ApoAlert DNA fragmentation kit (Clonetech, Mountain View, CA). The tissue was permeabilized in ice-cold NaCitrate buffer (0.1%NaCitrate, 0.1% Triton X-100) for 2 minutes. TdT reaction was performed at 37°C for 1 hour per manufacturer’s suggestion with the exception of using biotinylated dNTPs (New England Biolabs, Ipswich, MA), followed by AlexaFluor-conjugated StrepAvidin labeling (Molecular Probes) and nuclear stain with DAPI. Quantification of the number of TUNEL-positive cells was performed in the midbrain and hindbrain region in both

### Web Resources

The URLs for data presented herein are as follows:

PyMOL, https://www.pymol.org/

NHBLI Exome Sequencing Project, http://evs.gs.washington.edu/EVS/

ExAC database, http://exac.broadinstitute.org/

Golden Helix GenomeBrowse visualization tool (Version 2.0), http://www.goldenhelix.com

## Supporting Information

S1 Fig Purified RING domain of WT and C846G variant of VPS11. Peptides coding for VPS11 RING domain (amino acids 821–860, ~5 kDa) have been produced and purified has mentioned in Materials and Methods. Coomassie Blue stained gel shows the amount of the remaining proteins on glutathione-beads after protease cleavage and the purity of each elution 1, 2 and 3 of the WT (155, 95 and 60 μM respectively) and C846G (95, 56, 33 μM respectively) RING domain fragments used during CD experiments.
(TIF)

S2 Fig Quantification of Mbp staining intensity showed a significant reduction in *vps11(plt)* mutants at 7 dpf. The method uses NIH image J to measure the intensity of the signal in a given area. It measures the intensity of an area with no signal and subtracts this out as background. That gives the final value in an arbitrary unit of fluorescent intensity. Quantification of the intensity of the Mbp staining showed a significant reduction in vps11(plt) mutants at 7 dpf when compared with wild-type siblings (38% of control level expression; p<0.05; N = 5. In Fig 6).
(TIF)

S3 Fig Significant cell death in the CNS of zebrafish vps11(plt) mutants. Activated Caspase-3 was performed on cryosectioned CNS tissue from *vps11(plt)* mutants and wild-type siblings at 7 days post-fertilization (dpf). A) Wild-type hindbrain shows minimal apoptotic cells (arrow). B) Many apoptotic cells were observed in the *vps11(plt)* mutants. C) Quantification of the average number of Caspase-3+ cells observed in the hindbrain of *vps11(plt)* mutants and wild-type siblings at 7 dpf. Asterisk indicates significantly different from control.
(TIF)
