# Supplementary figures and images for: A Founder Mutation in VPS11 Causes an Autosomal Recessive Leukoencephalopathy Linked to Autophagic Defects
Source: PLoS Genet. 2016 Apr 27;12(4):e1005848. doi: 10.1371/journal.pgen.1005848 (PMC4847778; doi:10.1371/journal.pgen.1005848)

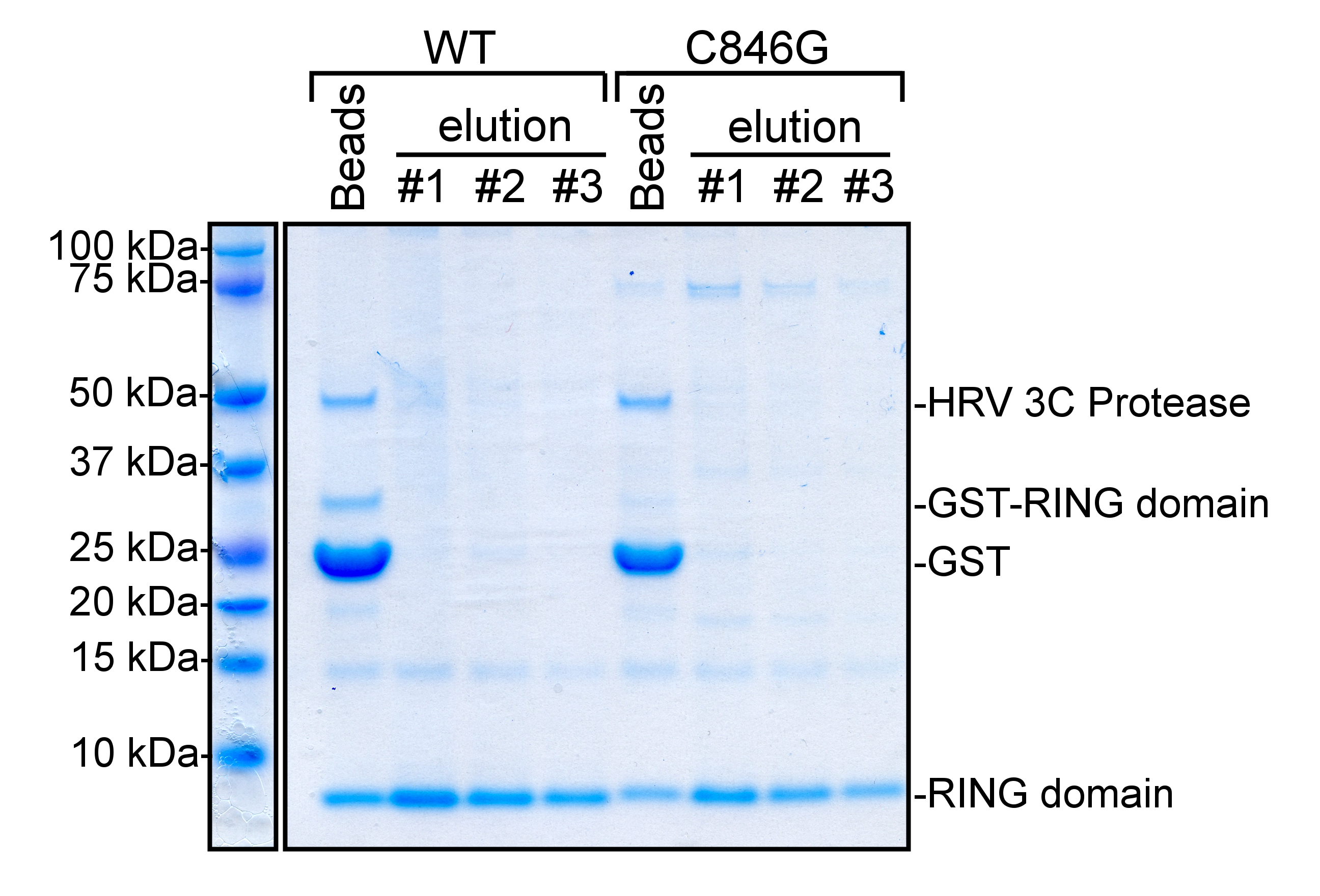

Supplement: S1 Fig — Peptides coding for VPS11 RING domain (amino acids 821–860, ~5 kDa) have been produced and purified has mentioned in Materials and Methods. Coomassie Blue stained gel shows the amount of the remaining proteins on glutathione-beads after protease cleavage and the purity of each elution 1, 2 and 3 of the WT (155, 95 and 60 μM respectively) and C846G (95, 56, 33 μM respectively) RING domain fragments used during CD experiments. (TIF) [file pgen.1005848.s001.tif]

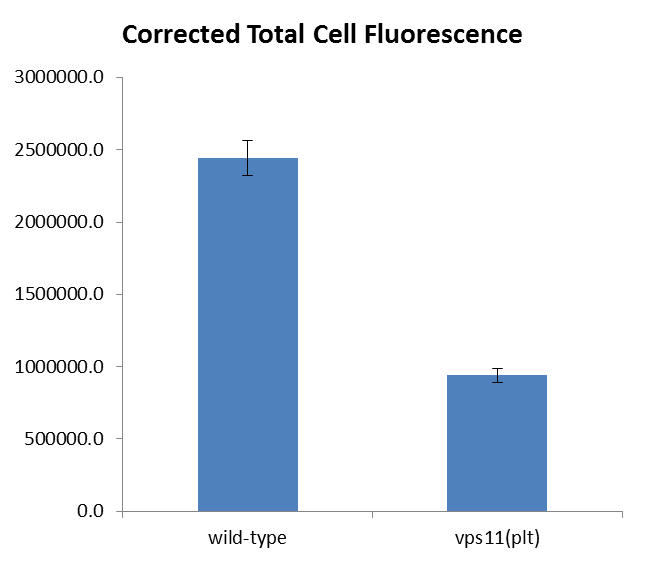

Supplement: S2 Fig — The method uses NIH image J to measure the intensity of the signal in a given area. It measures the intensity of an area with no signal and subtracts this out as background. That gives the final value in an arbitrary unit of fluorescent intensity. Quantification of the intensity of the Mbp staining showed a significant reduction in vps11(plt) mutants at 7 dpf when compared with wild-type siblings (38% of control level expression; p<0.05; N = 5. In Fig 6). (TIF) [file pgen.1005848.s002.tif]

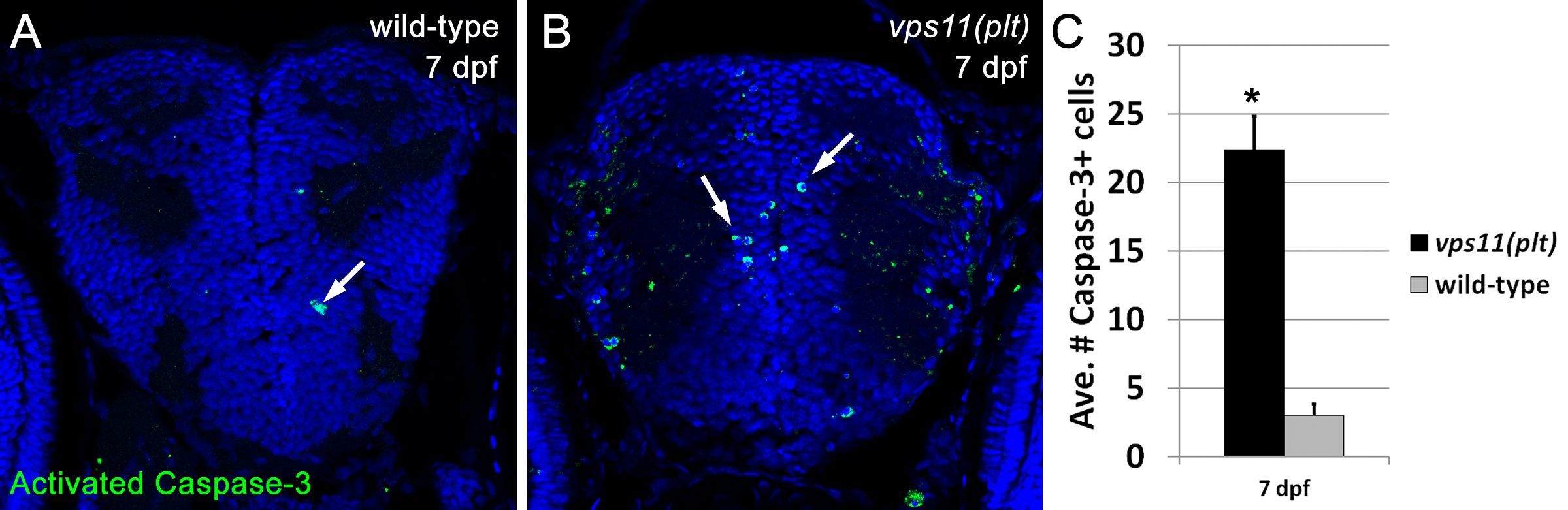

Supplement: S3 Fig — Activated Caspase-3 was performed on cryosectioned CNS tissue from vps11(plt) mutants and wild-type siblings at 7 days post-fertilization (dpf). A) Wild-type hindbrain shows minimal apoptotic cells (arrow). B) Many apoptotic cells were observed in the vps11(plt) mutants. C) Quantification of the average number of Caspase-3+ cells observed in the hindbrain of vps11(plt) mutants and wild-type siblings at 7 dpf. Asterisk indicates significantly different from control. (TIF) [file pgen.1005848.s003.tif]
